# Temporal evolution of hydroclimatic teleconnection and a time-varying model for long-lead prediction of Indian summer monsoon rainfall

**DOI:** 10.1038/s41598-018-28972-z

**Published:** 2018-07-17

**Authors:** Riya Dutta, Rajib Maity

**Affiliations:** Department of Civil Engineering, Indian Institute of Technology Kharagpur, Kharagpur, 721302, West Bengal India

## Abstract

Several cases of failure in the prediction of Indian Summer Monsoon Rainfall (ISMR) are the major concern for long-lead prediction. We propose that this is due to the temporal evolution of association/linkage (inherent concept of temporal networks) with various factors and climatic indices across the globe, such as El Niño-Southern Oscillation (ENSO), Equatorial Indian Ocean Oscillation (EQUINOO), Atlantic Multidecadal Oscillation (AMO), North Atlantic Oscillation (NAO), Pacific Decadal Oscillation (PDO) etc. Static models establish time-invariant (permanent) connections between such indices (predictors) and predictand (ISMR), whereas we hypothesize that such systems are temporally varying in nature. Considering hydroclimatic teleconnection with two major climate indices, ENSO and EQUINOO, we showed that the temporal persistence of the association is as low as three years. As an application of this concept, a statistical time-varying model is developed and the prediction performance is compared against its static counterpart (time-invariant model). The proposed approach is able to capture the ISMR anomalies and successfully predicts the severe drought years too. Specifically, 64% more accurate performance (in terms of RMSE) is achievable by the recommended time-varying approach as compared to existing time-invariant concepts.

## Introduction

Spatio-temporal variability of rainfall has significant economic and societal impacts, particularly for agriculture based countries. For instance, India receives more than 80% annual rainfall in four monsoon months (June-September) and substantial fluctuations are noticed in the annual food grain production due to the vagaries of monsoon. The association can be stated by the fact that the dips (such as 2002 and 2014) and peaks (such as 2013 and 2016) in the food grain production correspond to below normal and above normal monsoon rainfall respectively^1^. Naturally, considerable efforts including long-lead monsoon prediction needs to be made towards identifying and adopting strategies to deal with crisis situations. Due to considerable complexity in the evolution of ISMR, the long-lead prediction remains as a challenging task, particularly in a changing climate^2–4^. A large number of studies have analyzed the inter-annual variability of ISMR^5^; however the long-term climate fluctuations which modulate such variability are still not clear.

The long-range forecasting of ISMR was started more than a century ago. These are broadly grouped into statistical^6–18^ and dynamical^19–25^ forecasting approaches. Despite advancement in physical understanding and development of advanced statistical models the forecast failed in recent years too. Some major issues and inherent problems in the statistical models such as variation in the predictor-predictand relationship over time and conditional dependence among the predictors shows the necessity for constant scrutiny and update in the models^2,12,26–29^. The changes can be brought out in different ways, such as the use of new predictors, changing the combination of the predictors or lags, updating the model parameters, etc.

Predictor selection is an important aspect of statistical modeling and two climatic indices strongly influencing the variability of ISMR are El Niño-Southern Oscillation (ENSO) and Equatorial Indian Ocean Oscillation (EQUINOO)^10,13,15,30–32^. The concept of utilization of ENSO and EQUINOO as predictor lies in the hydroclimatic teleconnection^33,34^. However, we hypothesize that the nature of such association varies considerably with time and must be considered for consistency in long-lead prediction. The above and below normal summer monsoon rainfall from 1958 to 2003 are associated with favorable (unfavorable) phases of both the climatic indices^10^. In fact, inter-annual variation of ISMR is concurrently associated with ENSO and EQUINOO, for e.g. the impacts of El Niño during 1997 and 2002 were neutralized by negative/positive phases of EQUINOO^31^.

Though the statistical models used at present by IMD strongly acknowledge the association of large scale coupled atmospheric-oceanic circulation from tropical Pacific (e.g., ENSO) and tropical Indian Ocean (e.g., Equatorial South East Indian Ocean SST anomaly) with ISMR^35^, forecasts have failed in many recent years. The reason was considered to be weakening and or evolving nature of association between climatic indices and ISMR^26,36–38^. Continuous occurrence of such events over the years shows that the association between ISMR (predictand) and the climatic indices (predictor/input variables) varies with time. Thus, long-lead seasonal prediction of ISMR is required to consider two important issues – (i) identifying the most influencing predictors with appropriate lags and (ii) identifying the time-varying nature of predictor-predictand association. These form the motivation of this study. Conditional independence among the predictors is utilized to address the former issue and time-varying characteristics of the proposed model addresses the latter issue. Thus, the objective of this study is to develop a long-lead, time-varying statistical model for the prediction of ISMR along with uncertainty quantification. The potential of using time-varying approach is contrasted against classical time-invariant approach.

## Results

### Model development and selection of optimum time horizon

**Figure 1:**
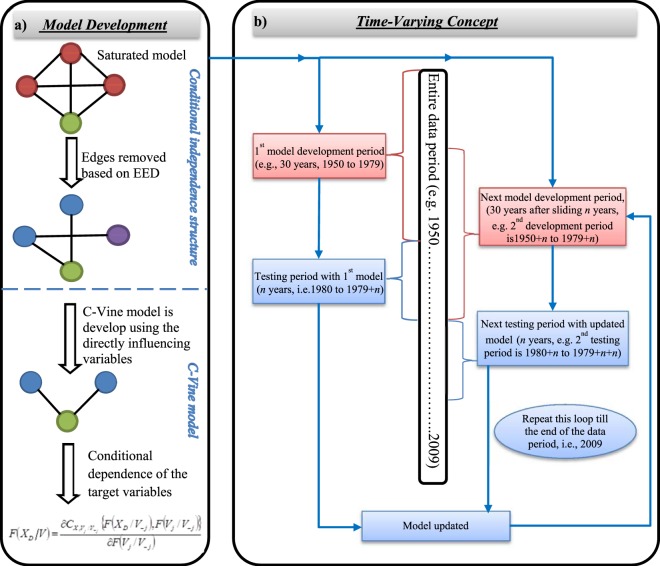
Methodological concept and flowchart. (**a**) Model development involves two major steps – development of the conditional independence structure and development of the C-Vine copula model. The conditional independence structure is utilized for identification of the potential predictors. In this process, initially a fully saturated model is considered where the red nodes signify the predictors and the green nodes signify the target variable. After eliminating the insignificant edges (at 95% significance level), the blue nodes designate the parents of the target variable and the purple node designates conditionally independent variables. Next, the C-Vine copula model is utilized to evaluate the conditional dependence of the target variable given the potential predictors (directly influencing variables). (**b**) The time-varying concept utilizes a series of moving model development periods that utilizes the concept shown in a. The time-varying concept is incorporated in the model by sliding the model development period by *n* years (optimum prediction horizon) and updating the input variables and the model parameters (Table 1).

**Figure 2:**
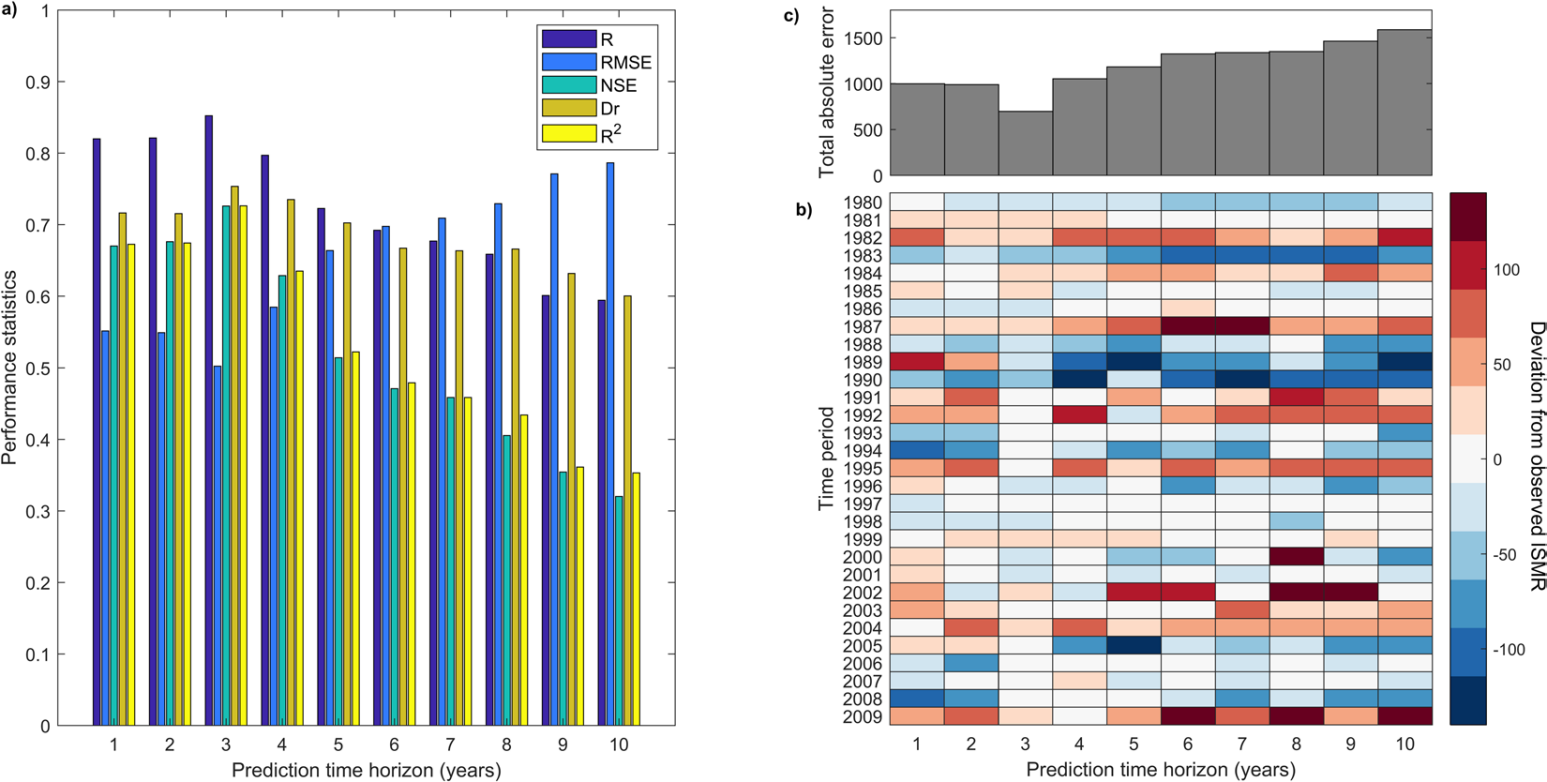
Selection of optimum time horizon for the time-varying model. (**a**) ISMR prediction during the testing period (1980–2009) is carried using the time-varying C-Vine model considering the prediction time horizons of 1 to 10 years for the selection of the optimal time horizon. A grouped bar-plot showing different performance statistics for the predicted results using each time horizon clearly depict 3 years as the optimal prediction time horizon, (**b**) a heat map showing the year-wise deviation of the predicted values from observed ISMR for each time horizon and entire model testing period, and (**c**) the map is associated to the total error for the entire testing period, which shows prediction time horizon of 3 years is optimum. As the prediction time horizon increases beyond 3 years, the value of error increases indicating the decreasing temporal persistence of the identified model.

Methodological outline and concept of time-varying approach is shown in Fig. 1. Details of mathematical steps and discussion are provided later in methodology section. At the outset, optimum prediction time horizon (*n* years) has to be decided for the development of the time-varying model. Towards this a range of prediction time horizons, starting from 1 to 10 years are utilized and the model development period is considered as a moving 30 years window. The conditional independence structure is used to obtain the potential predictors, listed in Table 1, for a particular model development period and the prediction model is developed by employing conditional dependence using C-Vine copula approach. The analysis is repeated for each prediction time horizons and the results are compared for identification of the optimum value of *n*. The performance statistics namely Correlation coefficient (R), Root Mean Square Error (RMSE), Index of agreement (Dr), Nash-Sutcliffe Efficiency (NSE) and Coefficient of determination (R^2^), comparing the observed and the predicted ISMR for different prediction time horizons are shown in Fig. 2a. A heat map depicting the deviation of predicted values from observed ISMR for each time horizon (1, 2… 10 years) during entire model testing period (1980–2009) is shown in Fig. 2b. As expected, with an increase in the prediction time horizon the model performance becomes poorer as it is unable to capture the variations in association between the climatic indices and ISMR, which is true for prediction time horizon beyond 3 years. On the other hand, prediction time horizon shorter than 3 years does not yield significant improvement in model performance. Moreover, associating with the heat map, the total absolute error for the entire testing period associated with each time horizon (Fig. 2c) depicts that minimum error is obtained for prediction time horizon of 3 years. Thus, the optimum value of *n* for the time-varying model is considered as 3 years. In other words, association between predictor set of climatic indices and ISMR is recommended to check and update the model parameter after every 3 years.

**Table 1:** Details of the model development period, testing period and the probabilistic models developed from the dependence structure for the respective time period using optimal time horizon of 3 years.

Sl. No	Model Development Period	Model Testing Period	Probabilistic Model with Inputs
1	1950–1979	1980–1982	$f(y/E{n}_{9},E{q}_{10})$
2	1953–1982	1983–1985	$f(y/E{n}_{9},E{q}_{10})$
3	1956–1985	1986–1988	$f(y/E{n}_{8},E{n}_{9},E{q}_{10})$
4	1959–1988	1989–1991	$f(y/E{n}_{8},E{n}_{9},E{n}_{10},E{n}_{11},E{n}_{12},E{n}_{13},E{q}_{11})$
5	1962–1991	1992–1994	$f(y/E{n}_{8},E{n}_{9},E{n}_{10},E{n}_{11},E{n}_{12},E{n}_{13},E{q}_{11})$
6	1965–1994	1995–1997	$f(y/E{n}_{8},E{n}_{9},E{n}_{10},E{n}_{11},E{q}_{11})$
7	1968–1997	1998–2000	$f(y/E{n}_{8},E{n}_{9},E{q}_{11})$
8	1971–2000	2001–2003	$f(y/E{n}_{8},E{n}_{9},E{n}_{10},E{n}_{11},E{q}_{10},E{q}_{11})$
9	1974–2003	2004–2006	$f(y/E{n}_{8},E{n}_{9},E{n}_{10},E{n}_{11},E{n}_{12},E{q}_{10,}E{q}_{11})$
10	1977–2006	2007–2009	$f(y/E{n}_{8},E{n}_{9},E{n}_{10},E{n}_{11},E{n}_{12},E{n}_{13},E{q}_{10},E{q}_{11})$

Considering an optimal time horizon of 3 years, successive model development periods are 1950–1979, 1953–1982, 1956–1985, …, 1977–2006, and the corresponding testing periods are 1980–1982, 1983–1985, 1986–1988, …, 2007–2009 respectively. As a typical example, the conditional independence structure for the first model development period (1950–1979) is shown in Supplementary Fig. S1. It is noticed that the variation of ISMR is directly dependent on the August (9^th^ lag) ENSO $(E{n}_{9})$ and July (10^th^ lag) EQUINOO $(E{q}_{10})$ of the previous year and thereby stated as the potential predictors. Thus probabilistic model is developed using the potential predictors and the corresponding model is $f(y/E{n}_{9},E{q}_{10})$. Similar conditional independence structures have been developed for all the model development periods using the directly influencing predictors (Table 1). It can be noticed, that the input variables and corresponding model structure is getting modified gradually over time, indicating the necessity of time-varying model.

### Time-varying association of the climatic indices and ISMR

**Figure 3:**
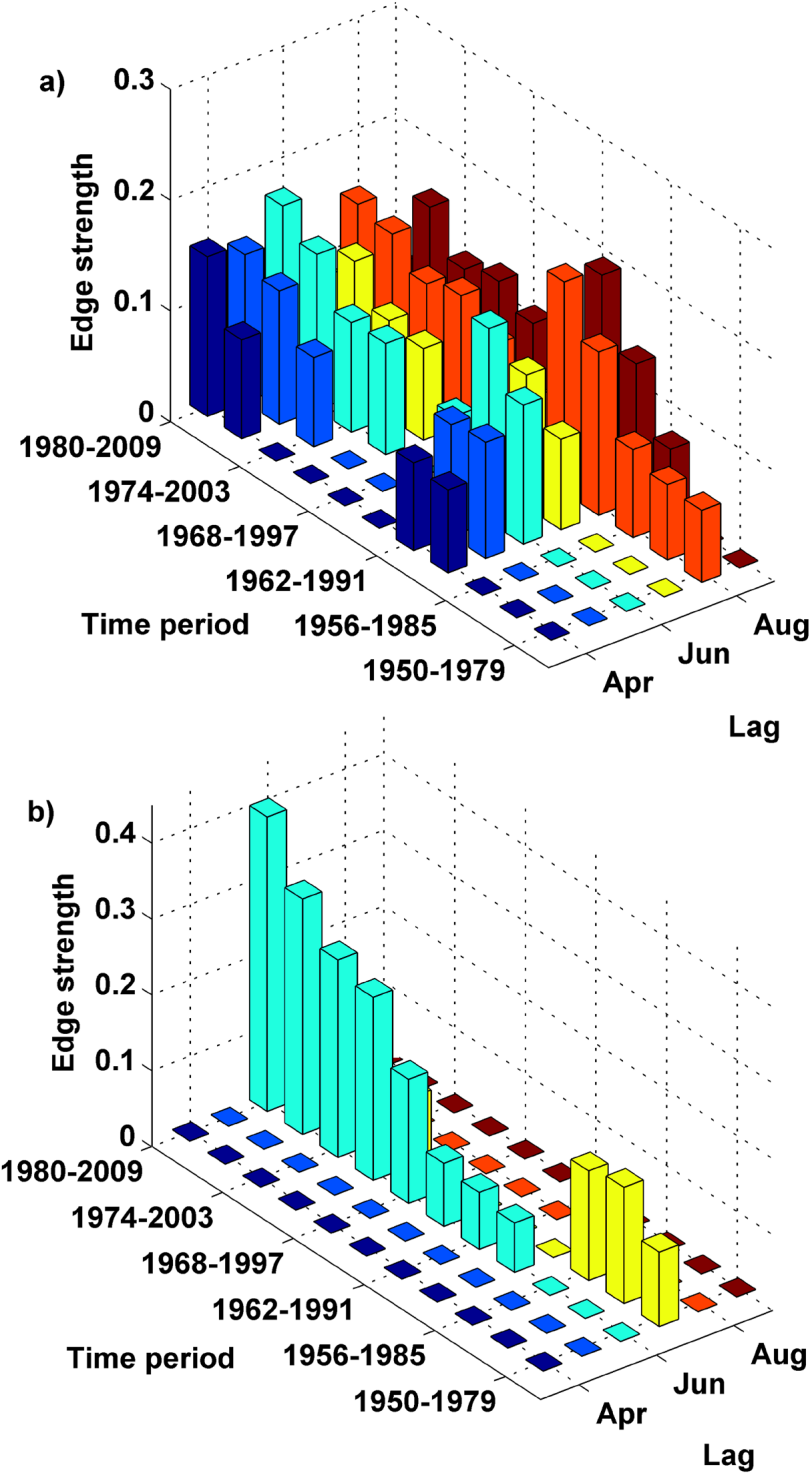
The significant edges between the climatic indices and ISMR for time horizon of 3 years. (**a**) The presence or absence of association between the different lags of ENSO and ISMR is investigated through their respective edge strengths. The significant edges and the corresponding edge strength for a particular lag of ENSO with ISMR is shown by the bar plot. It is observed that the August (9^th^ lag) ENSO of the previous year appears in 1950–1979, however with very low strength of association. The remaining five lags (8^th^, 10^th^, 11^th^, 12^th^ and 13^th^ lag) appear in 1959–1988 and show an increasing strength of association till 1962–1991 and then gradually reduce. Again the edges appear around 1971–2000 and the edge strength for all the lags (8^th^ to 13^th^ lag) shows an increasing association till 2009, and (**b**) the significant edges and the corresponding edge strength for a particular lag of EQUINOO with ISMR is shown by the bar plot. It can be observed that only July and June (10^th^ and 11^th^ lags) EQUINOO of the previous year, show significant association with ISMR. The July EQUINOO of previous year appears in the time period 1959–1988, right around the years lags of ENSO showed an appearance, and is consistently showing an increase in the edge strength till 2009. The June EQUINOO of previous year also shows significant edge strength right from 1950, then gradually reduces, again reappears around 1971 and again disappears by 2009.

After identification of the potential predictors, the time variability of their effectiveness can be studied using the edge strength. The temporal variation of association between ISMR and the climate indices at different lags are shown in Fig. 3 (3-D plots showing only the significant edge strength for both ENSO and EQUINOO), Supplementary Fig. S2 (ENSO), and Supplementary Fig. S3 (EQUINOO) along with the significance threshold of Edge Exclusion Deviance (EED) >3.84. The analysis was carried out using April (1^st^ lag) ENSO and EQUINOO of present year to April (13^th^ lag) ENSO and EQUINOO of the previous year. However, significant edge strength were found only in the range of September to April (8^th^ to 13^th^ lag) ENSO and EQUINOO of the previous year. Thus, the above mentioned figures depict these lags only. The plots show the temporal variation in association of September to April (8^th^ to 13^th^ lag) ENSO and EQUINOO of the previous year, the time when these were found statistically significant. Supplementary Fig. S2 shows an increasing trend in the strength of association between different lags of ENSO and ISMR from 1962 to 1991, which reaches a peak around 1991. Then the strength of association drastically reduces for most of the lags and again increases gradually till 2009. Supplementary Fig. S3, shows that April, May, August and September EQUINOO of previous year do not have any significant edges with ISMR, that is the strength of association is either very low or nil. However, July and June EQUINOO of previous year shows significant association, where July EQUINOO of previous year shows an increasing trend and June EQUINOO of previous year shows a decreasing trend. The 3-D plots in Fig. 3 explicitly show the appearance and disappearance of different lags of ENSO and EQUINOO. The appearance (disappearance) of the bars in these figures indicates the significant (insignificant) edge strength for that particular lag at 95% significance level. Such variation in the strength of association may be the major source of inconsistency in seasonal forecasting.

### Performance and comparison of time-varying C-Vine model

In general, the time-varying association suggests that the time-invariant set of predictors may suffer from consistency in performance. To investigate this fact, the performance of time-varying approach and time-invariant approaches are compared.

**Figure 4:**
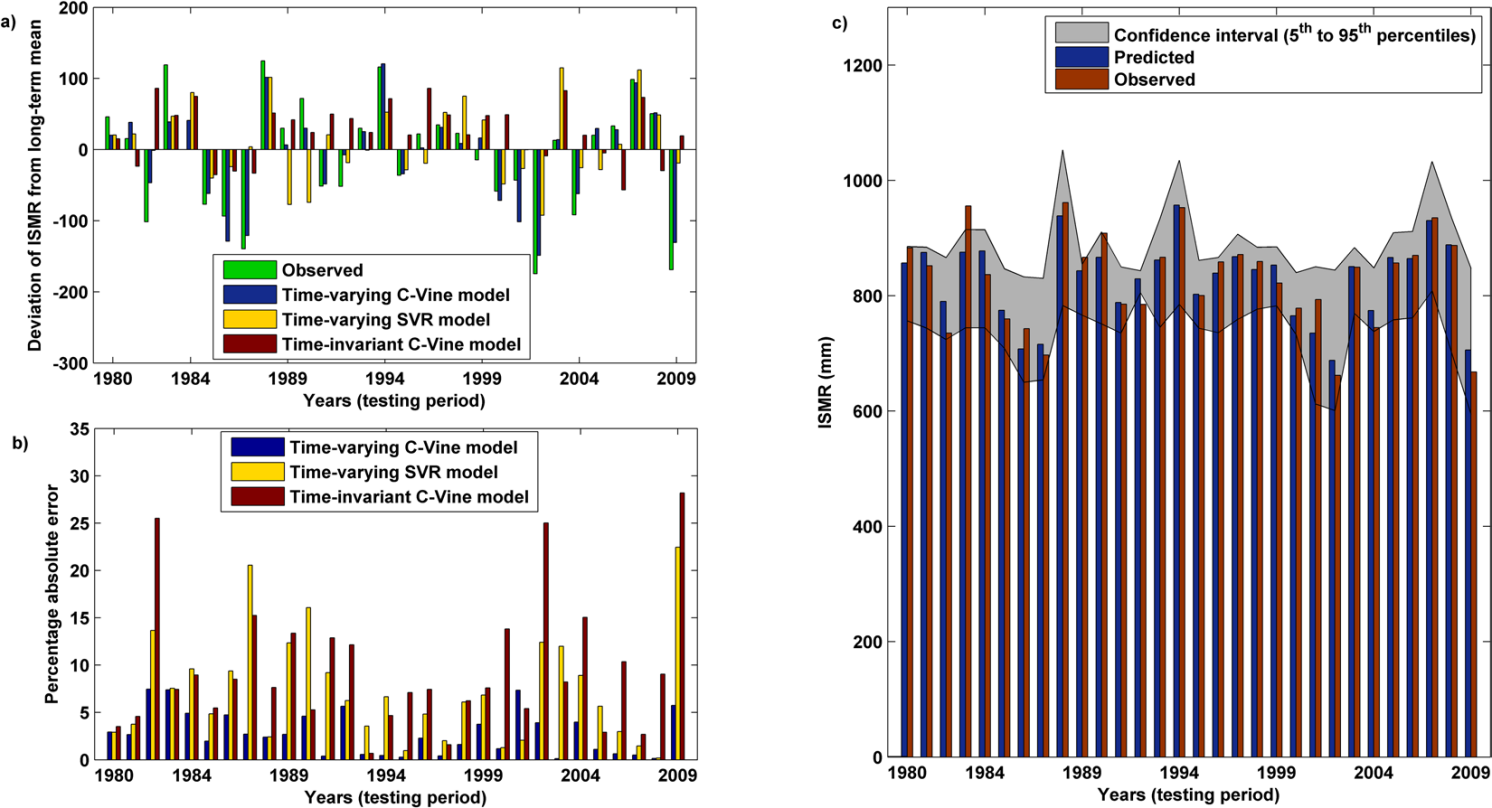
Comparison of the performance between time-varying and time-invariant approaches. (**a**) Observed and predicted rainfall anomaly (deviation from long-term mean) obtained using the time-varying C-Vine model, time-varying SVR model and time-invariant C-Vine model. For clarity time-invariant SVR model is not included which performs much poorer (Table 2), (**b**) the percentage absolute error resulted from aforementioned three prediction models. The time-varying C-Vine model is found to be the best performing model, and (**c**) comparison of the observed and predicted (50^th^ percentile) ISMR using the time-varying C-Vine model. The uncertainty band of the predicted ISMR is shown as an envelope where the lower and the upper limits corresponds to the 5^th^ and 95^th^ percentile respectively.

To assess the improvement gained through the time-varying approach as compared to time-invariant approach, four models are used – Time-varying C-Vine model, Time-varying Support Vector Regression (SVR) model, Time-invariant C-Vine model and Time-invariant SVR model. In case of time-invariant approaches, the model developed for time period 1950–1979 is used for prediction of ISMR for the entire testing period 1980–2009. The relative efficacy of first three models, in capturing the variation in anomalous ISMR (deviation from the long-term mean), is shown in Fig. 4a. The corresponding percentage errors obtained using these models are shown in Fig. 4b. Absolute errors are shown for easy comparison. The actual values of error percentage are given in Supplementary Table S1. It is noticed that the time-invariant SVR model performs very poorly and excluded from Fig. 4a,b for clarity. In general, the time-varying models are found to yield more accurate results, as compared to the time-invariant model, rightly capturing the nature/behavior of the recorded anomalous rainfall values in almost all the years during the testing period (Fig. 4a,b). The significantly above normal (e.g., 1988, 1994) and below normal (e.g., 1986, 1987, 2002, 2004, and 2009) ISMR are very well captured. Thus, the results of the proposed approach suggest that the time-varying model used in this study appropriately predicts the ISMR anomalies including major drought years, such as 2002, 2004 and 2009.

The comparison is also carried out between the predicted values obtained from different models and the actual values of ISMR during the testing period through scatter plot (Supplementary Fig. S4) and performance statistics. The performance statistics obtained using time-varying C-Vine model is superior in comparison to its time-invariant counterpart as well as time-varying and time-invariant SVR model (Table 2). Thus, the C-Vine copula based time-varying model is able to provide the most precise long-lead (almost a year ahead) ISMR anomaly pattern. In general, the time-varying concept provides better results.

**Table 2:** Model performances during the testing period (1980–2009).

Performance Statistics	Model Used			
	Time-varying C-Vine	Time-invariant C-Vine	Time-varying SVR	Time-invariant SVR
R	0.85	0.28	0.69	0.12
RMSE	30.16	84.74	58.28	103.88
NSE	0.72	0.02	0.42	0.01
Dr	0.75	0.53	0.65	0.36
R^2^	0.72	0.07	0.48	0.01

In general, the time-varying concept provides better results and time-varying C-Vine model provides best results.

Time-varying association has been identified as one of the major issues, however it is also true that the complex nature of the association needs an advance statistical model, such as C-Vine copula based approach as used in this study. This is established through the inferior performance of time-varying SVR model as compared to time-varying C-Vine model (Fig. 4a,b). Minimum error in case of time-varying C-Vine model is confirmed for all the years in the testing period except 2001, when the error with time-varying C-Vine model was found to be 7%. For this particular year and only for this year, time-invariant C-Vine model shows better performance with 5% error. However, time-varying SVR model yields a 2% error in 2001. Thus, in general, the time-varying concept provides better results for 2001 also. Slightly poorer performance of time-varying C-Vine model could not be explicitly pointed out. However, in general, we also agree that consideration of other factors (discussed later) may improve the results in general including the stray performances as noticed in 2001.

The C-Vine copula based approach also provides conditional uncertainty quantification, which is another useful information. It is due to probabilistic nature of prediction which is obtained from the conditional distribution, conditioned on the potential inputs. Figure 4c shows the confidence interval (uncertainty band) of the predicted ISMR with the lower and the upper limits as 5^th^ and 95^th^ percentile respectively, and the 50^th^ percentile values are compared with the observed values. It is noticed that most of the observed values are captured within this range.

## Discussion and Conclusion

The problem of long-lead prediction of ISMR has been addressed, through a time-varying model, which uses lagged large scale climatic indices (ENSO and EQUINOO) as the predictors. Previously 1997, 2002, 2004 and 2009 were the examples of failure in ISMR prediction^4,10,26,39^. The year 1997 was expected to be a drought due to a huge El Niño event year however turned out to be normal and the years 2002, 2004 and 2009 were not expected but turned out to be severe drought years. Weakening relationship, unaccounted influence of Indian Ocean Dipole, and non-stationarity are some of causes listed in the literature and new analyses are attempted in several studies^26,35–38,40^. Our analysis indicates the possible cause could be the absence of temporal persistence in the network structure of the prediction model. That means the temporal links owing to hydroclimatic teleconnections changes over time. In this context, temporal networks, that changes over time, have proven to be advantageous compared to their static counterparts that are characterized by permanent connections between variables^29,41^. In our study, inherent concept of the temporal networks is used that is the network structure itself changes over time. Towards this, identification of the directly influencing predictors and their respective lags poses a challenge in the field of long-lead seasonal prediction of ISMR as the number of influencing variables is high. The conditional independence structure is employed to identify the association among the different lags of climatic indices and ISMR. It can be noticed, that the input variables and corresponding model structure is getting modified gradually over time, indicating the necessity of time-varying model. The time-varying nature of the association throttles the consistency of any prediction model and can be addressed by considering the time-variation in the predictand–predictor relationship as utilized in the proposed model. Temporal networks also emphasize on the concept of identification of the optimal prediction time horizon and updating the predictors and the parameters of the model for each moving window^29^. Literature states that optimization of the prediction time horizon is vital as larger time horizons are unable to capture the temporal variation in associations and tends to a more static network and shorter time horizons may provide erroneous results. In this study, it is found that the model should be updated after an optimum prediction time horizon of 3 years after which the model and the input set needs to be updated.

The lead time of prediction for the time-varying model is 8 months, which enables us to predict the nature of ISMR for the following summer monsoon season at the end of the present monsoon season. In brief, time-varying, C-Vine copula based approach provides less than 5% error in most of the years and less than 10% error in all the years during the testing period (1980–2009) (Fig. 4b). Thus, the consistent performance is achieved following the proposed approach.

The performance of time-varying approach is found to yield more accurate results, as compared to the time-invariant model, rightly capturing the anomalies of ISMR. To be specific, 64% more accurate performance (in terms of RMSE) is obtained by the proposed time-varying model as compared to time-invariant C-Vine model. Further, among the time-varying approaches, C-Vine based model is found to provide 21.8% more accurate performance in terms of RMSE as compared to SVR based approach.

Finally, we like to state that this study uses two major climatic indices, however many other large scale indices namely, Atlantic Multidecadal Oscillation (AMO), North Atlantic Ocsillation (NAO), Pacific Decadal Oscillation (PDO), El Niño Modoki Index (EMI) etc. influence the ISMR. Using the time-varying concept, these climatic indices can be also used in addition to ENSO and EQUINOO. Moreover similar studies can be carried out at finer spatial scale to study the time-varying association of the climatic indices and other factors with the variability of regional rainfall.

## Data and Methodology

### Data Used

The following data sets for the period 1950–2009, are used in this study: (i) ISMR obtained from Indian Institute of Tropical Meteorology (available at www.tropmet.res.in), (ii) SST anomaly over Niño3.4 region (120°–170°W, 5°S–5°N) obtained from climate analysis section, National Center for Atmospheric Research, USA (available at www.cgd.ucar.edu) and (iii) surface wind data^42^ from National Center for Environmental Prediction (available at www.cdc.noaa.gov). Rainfall anomaly values are obtained considering 1970–2000 as base period and EQUINOO^43^ index is computed as the negative of the zonal wind anomaly at surface in the equatorial Indian Ocean region (60°E–90°E, 2.5°S–2.5°N).

### Methodology

The overall methodology consists of several important aspects. First, it starts with the identification of the time-varying association of the predictors (ENSO and EQUINOO with several lags) with ISMR. However, there could be redundancy in information from different input variables, i.e., same information may be available from more than one variable. Hence, the predictors are selected based on the conditional independence structure between the pool input variables and the target variable (ISMR). Conditional independence structure helps to identify the variables that are directly or indirectly influencing the target variable. No influence of some variables is also identified, if such variable(s) exist (exists) in the pool of input variables. Indirectly influencing variables are known as conditionally independent for the target variable, i.e., given the directly influencing variables, such conditionally independent variables can be ignored from the set of predictors. Next, discarding all the independent and conditionally independent input variables, the prediction model is developed using C-Vine copula approach.

Secondly, a prediction time horizon (*n* years) after which the model needs to be updated to impart the time-varying characteristics needs to be identified. This is optimized in such a way that it should not be too long to miss the temporal variation in association and too short to avoid frequent updating. An optimum value of *n* years will ensure the best possible prediction results.

Finally, the time-varying model is compared with the existing time-invariant concept based model to study the benefit of the former. A flowchart showing the overall methodological concept is given in Fig. 1 and the mathematical formulations of all the steps are elaborated in the following sections.

### Predictor selection based on the conditional independence structure

The conditional independence among the input and target variables can be revealed through a graph, which is a mathematical object, *G* = (*V*, *E*), where *V* is a set of vertices or nodes (representing the variables) and *E* is a set of edges (representing the association among the variables). The identification of the conditional independence structure among the input variables (ENSO and EQUINOO with lag 1 to 13 months) and target variable is determined using the maximum likelihood approach^44^. In this approach, initially a fully interconnected graph structure (also referred to as a saturated model) is considered where all the nodes are connected to each other. Next, the Edge Exclusion Deviance (EED) is used for testing if an edge can be eliminated from the saturated model^44^. The formula for the calculation of EED is as follows,

1 $$EED=-\,N\,\mathrm{log}(1-cor{r}_{N}^{2}({X}_{i},\,{X}_{j}|{\rm{rest}}))$$

where *N* is the size of the sample and $cor{r}_{N}^{2}({X}_{i},{X}_{j}|{\rm{r}}{\rm{e}}{\rm{s}}{\rm{t}})$ is the partial correlation coefficient between any two random variables *X*_*i*_ and *X*_*j*_ given the rest. The statistics EED follows a chi-squared distribution, at 5% significance level with one degree of freedom (as one edge is removed at a time). The threshold of EED is 3.84, so the edges for which the EED is less than 3.84 are to be excluded. For application of this approach the data should follow normal distribution, else it can be transformed using some transformation methodology (e.g., Box and Cox transformation^45^).

To check the acceptability of the obtained independence structure at a particular confidence level a test statistic, known as the deviance, can be used. The generalized likelihood ratio test statistics, evaluated based on the observed sample variance and the estimated variance obtained from the independence structure is called the deviance of the model. The deviance can be evaluated as follows^44^,

2 $$dev=N\{tr(S{\hat{V}}^{-1})-\,\mathrm{log}\,\det (S{\hat{V}}^{-1})-K\}$$

where *S* is the variance matrix, $\hat{V}$ is the estimated variance matrix evaluated based on the number of edges removed from the model, *K* is the number of random variables and *N* is as stated before. The deviance (*dev*) follows an approximate chi-squared distribution with *d* degrees of freedom (where, *d* is the number of edges excluded from the saturated graph). Thus, p-value of the test statistics can be computed as $P({\chi }_{p}^{2} > dev)$. For this study the acceptable significance level is fixed at 0.05, i.e. the obtained conditional independence structure is acceptable if the p-value is higher than 0.05. In case the structure fails to meet the acceptability criteria, structure is to be modified with lesser number of edges removed from the saturated graph.

Whereas the conditional independence structure helps to identify the potential predictors, time variability of their potential is another important aspect. This is quantified through a statistical measure known as edge strength. Thus, the surviving edges of the conditional independence structure are investigated for their strength of association, also known as edge strength. The edge strength between two nodes (for a surviving edge) in the conditional independence structure can be calculated as follows^44^,

3 $$Inf({X}_{i}\coprod {X}_{j}|rest)=-\,\frac{1}{2}\,\mathrm{log}(1-cor{r}_{N}^{2}({X}_{i},{X}_{j}|rest))$$

where $Inf({X}_{i}\coprod {X}_{j}|rest)$ is the edge strength between *X*_i_ and *X*_j_ given rest. This is also known as divergence against conditional independence^44^. This information on edge strength is used to investigate the temporal evolution of association of a particular input with the ISMR.

### Development of time-varying association based model

First step towards the development of the time-varying model is to identify the optimum prediction horizon after which the model needs to be updated. Denoting this as *n* years, the time-varying association between the predictors and the predictand is captured by updating the potential predictor after each *n* years. Development of the time-varying association based model and optimization of the prediction time horizon to update the model are described as follows: a climatological time scale is recommended for any hydroclimatic model development and thus 30 years is used as model development period which is considered as a moving window in the time-varying approach. The concept is as follows: considering the prediction time horizon for updating the prediction model to be *n* years, the first model development period is considered from 1950 to 1979 and the model testing period is from 1980 to1980 + (*n* − 1). As the model is updated after *n* years the next model development period is shifted by *n* years. Thereby, the second model development period is considered from 1950 + *n* to 1979 + *n* and the model testing period is from (1979 + *n*) + 1 to (1979 + *n*) + 1+ (*n*−1). To identify the optimum prediction time horizon, the procedure is repeated for *n* = 1, 2, 3, …, 10 years. The model performance is noted in each case for the contiguous model testing periods during 1980–2009.

### Development of the prediction model

The probabilistic model is developed using the selected predictors identified through the conditional independence structure. Even after obtaining the structure, there could be multiple predictors directly associated with ISMR. Multivariate copulas, like nested copula or vine copula are the best choice to develop a multivariate probabilistic model. Among different alternatives in vine copulas, canonical vine (C-Vine) is used in this study to develop the probabilistic model. C-Vine can be used for prediction of a variable by a sequence of trees^46–50^. These trees are referred as C-Vines and the corresponding multivariate distribution is called C-Vine distribution. For a *D*-dimensional C-Vine (considering *D* − 1 number of predictors are selected based on the conditional independence structure), the first tree identifies (*D* − 1) pairs of variables whose distribution is modeled directly, utilizing the random variables. The second tree identifies (*D* − 2) pairs of variables whose distribution is conditional on a single variable evaluated by pair copula. This tree uses transformed variables based on the structure of the preceding tree. Proceeding, in this manner the final tree determines a single pair of variables conditional on the remaining variables. The analysis using C-Vine includes identifying the trees, its pair copula families and estimating their parameters.

Selection of each tree is based on a maximum spanning tree algorithm, where edge weights are chosen to reflect the dependencies. In this case, the absolute value of the empirical Kendall’s tau (${\hat{\tau }}_{i,j}$) (evaluated for two adjoining variables of the tree *X*_*i*_ and *X*_*j*_) is utilized as the edge weight and optimization problem is solved ($\max \sum _{edges\,{e}_{ij}\,\in \,in\,spanning\,tree}|{\hat{\tau }}_{ij}|$ where, a spanning tree is a tree on all nodes) for each tree^51^. Evaluation of the transformed variables (for selection of the subsequent trees after the first tree) requires estimation of the pair copula families and parameter estimation based on the conditioning variables. Considering *X*_*D*_ as the target variable and ${X}_{1},\,{X}_{2},\,\mathrm{...},\,{X}_{D-1}$ as the conditioning variables (predictors), the conditional distribution can be developed for a $(D-1)$ dimensional vector $V=({X}_{1},{X}_{2},\,\mathrm{...},{X}_{D-1})$ by applying the following recursive relationship‒

4 $$F({X}_{D}/V)=\frac{\partial {C}_{X,{V}_{j}/{V}_{-j}}\{F({X}_{D}/{V}_{-j}),F({V}_{j}/{V}_{-j})\}}{\partial F({V}_{j}/{V}_{-j})}$$

where ${V}_{j}(j=1,\,2,\,\mathrm{...},\,D-1)$ is an arbitrary component of $V$, and ${V}_{-j}=({X}_{1},\,{X}_{2}\,,\mathrm{...},\,{X}_{j-1},\,{X}_{j+1},\,\mathrm{...},\,{X}_{D-1})$ denotes the vector *V* excluding element *V*_*j*_. The bivariate copula function is specified by ${C}_{X,{V}_{j}/{V}_{-j}}$. The final tree can be utilized to evaluate the conditional dependence for prediction of the target variable given the input variables using the above equation.

## Electronic supplementary material

Supplementary Tables and Figures
