# Supplementary material for: Temporal evolution of hydroclimatic teleconnection and a time-varying model for long-lead prediction of Indian summer monsoon rainfall
Source: Sci Rep. 2018 Jul 17;8:10778. doi: 10.1038/s41598-018-28972-z (PMC6050344; doi:10.1038/s41598-018-28972-z)
Supplement: Supplementary file 1 — Supplementary Tables and Figures [file 41598_2018_28972_MOESM1_ESM.docx]

**Temporal evolution of hydroclimatic teleconnection and long-lead prediction of Indian summer monsoon rainfall using time varying model**

Riya Dutta and Rajib Maity

Department of Civil Engineering, Indian Institute of Technology Kharagpur, Kharagpur – 721302, West Bengal, India

**List of Supplementary Tables and Figures:**

Table S1: Percentage error for the results obtained during the model testing period (1980-2009) using the four models.

Figure S1: The conditional independence structure with edge strength among the climatic indices (En_i_ or Eq_j_) and ISMR (*Y*) for the time period of 1950-1979. The climate indices with recent lags are found to be completely independent (shaded region) whereas the indices with longer lags are found to be the potential predictors. The structure varies over time and recommended to update every three years (Ref. section 4 for details).

Figure S2: Time variation of edge strength between ISMR (predictand) and lags 8 to 13 of ENSO (predictors) obtained from the conditional independence structures along with significance threshold value (red line).

Figure S3: Time variation of edge strength between ISMR (predictand) and lags 8 to 13 of EQUINOO (predictors) obtained from the conditional independence structures along with significance threshold value (red line).

Figure S4: A scatter plot between the actual and predicted ISMR. (a) The plot obtained using the time-invariant SVR approach shows the most inferior performance, (b) the plot obtained using the time-invariant C-Vine approach also shows very inferior performance as the model is static in nature, (c) the plot obtained using time-varying SVR approach shows a comparatively better performance as the time-varying association of the climatic indices and ISMR is considered, and (d) time-varying C-Vine approach show the most superior results as vine copula rightly captures the complex association between ISMR and the climatic indices.

*Table S1: Percentage error for the results obtained during the model testing period (1980-2009) using the four models*

| Year | Deviation of the predicted ISMR from observed data (%) | | | |
| --- | --- | --- | --- | --- |
|  | Time-varying C-Vine | Time-varying SVR | Time-invariant C-Vine | Time-invariant SVR |
| 1980 | -2.94 | -2.91 | -3.51 | -5.05 |
| 1981 | 2.67 | 3.75 | -4.57 | 5.44 |
| 1982 | 7.47 | 13.66 | 25.50 | 27.78 |
| 1983 | -7.39 | -7.56 | -7.44 | -9.56 |
| 1984 | 4.91 | 9.61 | 8.97 | 9.40 |
| 1985 | 1.97 | 4.84 | 5.48 | 6.32 |
| 1986 | -4.74 | 9.39 | 8.52 | 10.02 |
| 1987 | 2.70 | 20.56 | 15.25 | 21.53 |
| 1988 | -2.41 | -2.42 | -7.65 | -8.46 |
| 1989 | -2.69 | -12.35 | 13.37 | -13.15 |
| 1990 | -4.61 | -16.07 | -5.27 | -19.29 |
| 1991 | 0.39 | 9.20 | 12.88 | 11.58 |
| 1992 | 5.66 | 6.25 | 12.15 | 13.41 |
| 1993 | -0.56 | -3.55 | -0.69 | -4.59 |
| 1994 | 0.45 | 6.67 | -4.67 | -7.88 |
| 1995 | 0.28 | 0.97 | 7.11 | 7.23 |
| 1996 | -2.28 | -4.83 | 7.45 | 10.59 |
| 1997 | -0.41 | 2.02 | 1.59 | 5.61 |
| 1998 | -1.63 | 6.10 | -6.24 | 8.53 |
| 1999 | 3.77 | 6.84 | 7.61 | 13.95 |
| 2000 | -1.18 | 1.29 | 13.81 | 16.47 |
| 2001 | -7.35 | 2.06 | 5.40 | -5.39 |
| 2002 | 3.91 | 12.42 | 25.02 | 26.45 |
| 2003 | 0.12 | 12.00 | 8.21 | 14.98 |
| 2004 | 3.99 | 8.92 | 15.05 | 18.42 |
| 2005 | 1.10 | -5.66 | -2.90 | -10.23 |
| 2006 | -0.63 | -2.97 | -10.35 | -15.22 |
| 2007 | -0.50 | 1.46 | -2.70 | 2.89 |
| 2008 | 0.14 | -0.19 | -9.04 | -11.54 |
| 2009 | 5.74 | 22.45 | 28.19 | 26.41 |


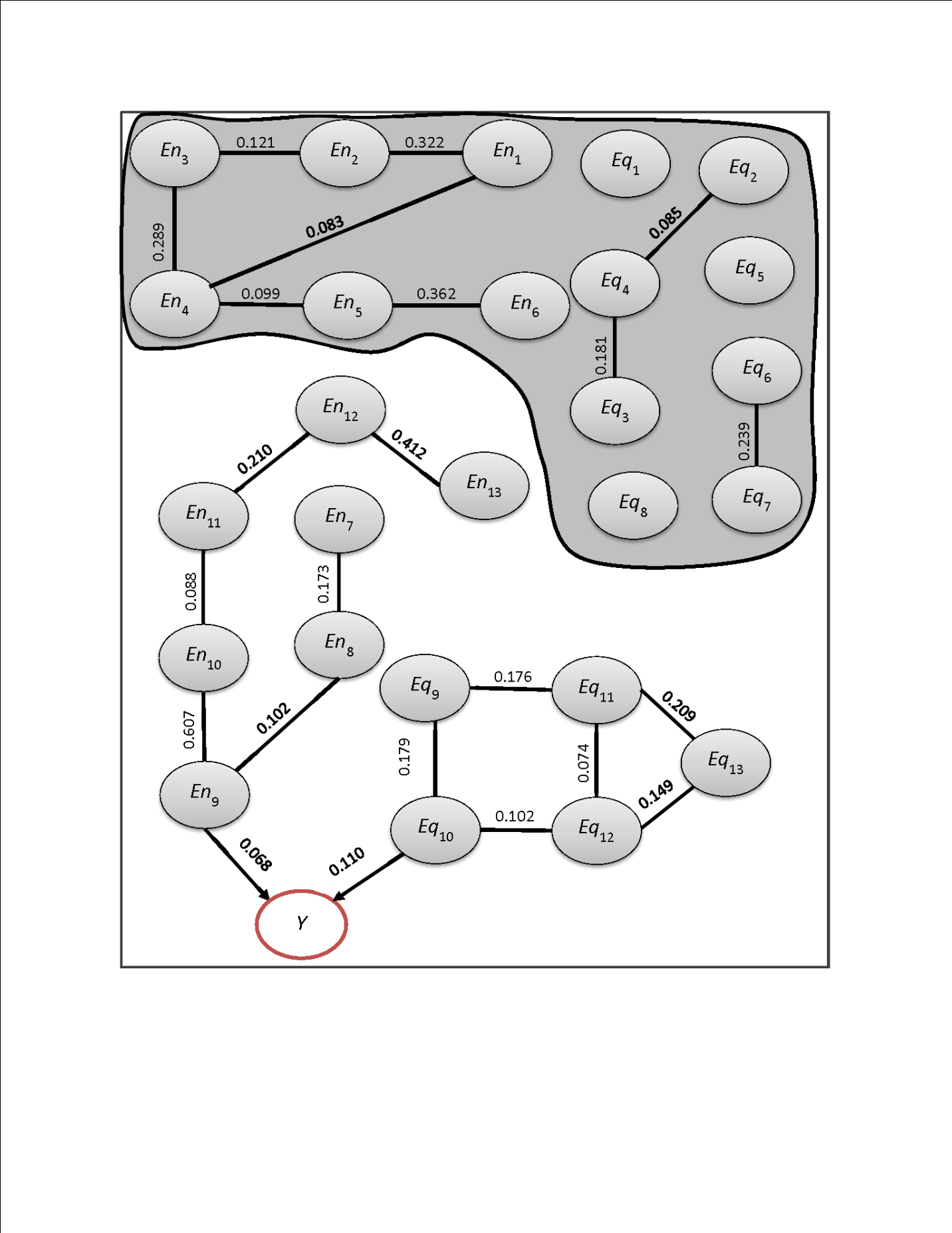


Figure S1: The conditional independence structure with edge strength among the climatic indices (En_i_ or Eq_j_) and ISMR (Y) for the time period of 1950-1979. The climate indices with recent lags are found to be completely independent (shaded region) whereas the indices with longer lags are found to be the potential predictors. The structure varies over time and recommended to update every three years (Ref. section 4 for details).


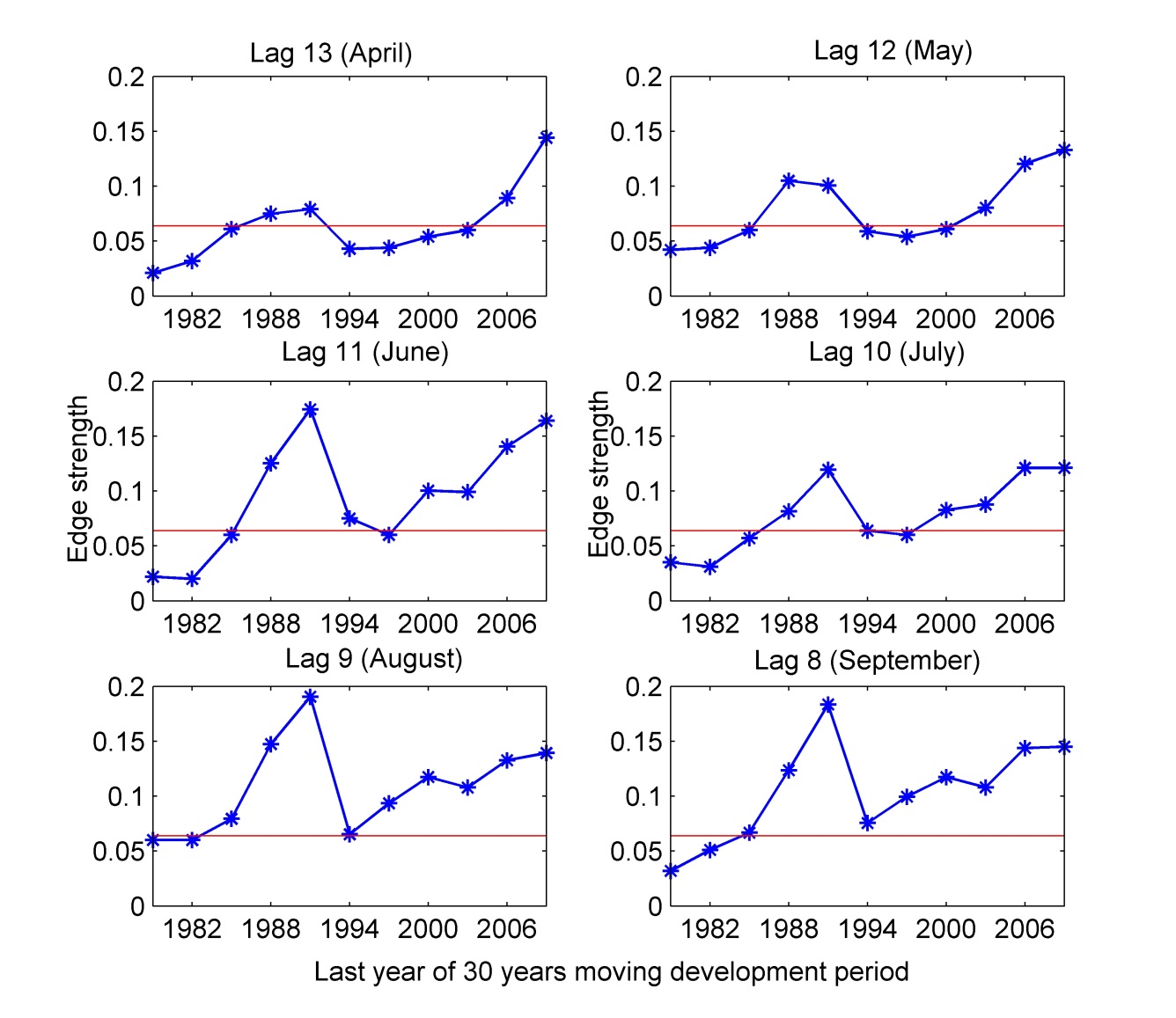


Figure S2: Time variation of edge strength between ISMR (predictand) and lags 8 to 13 of ENSO (predictors) obtained from the conditional independence structures along with significance threshold value (red line).


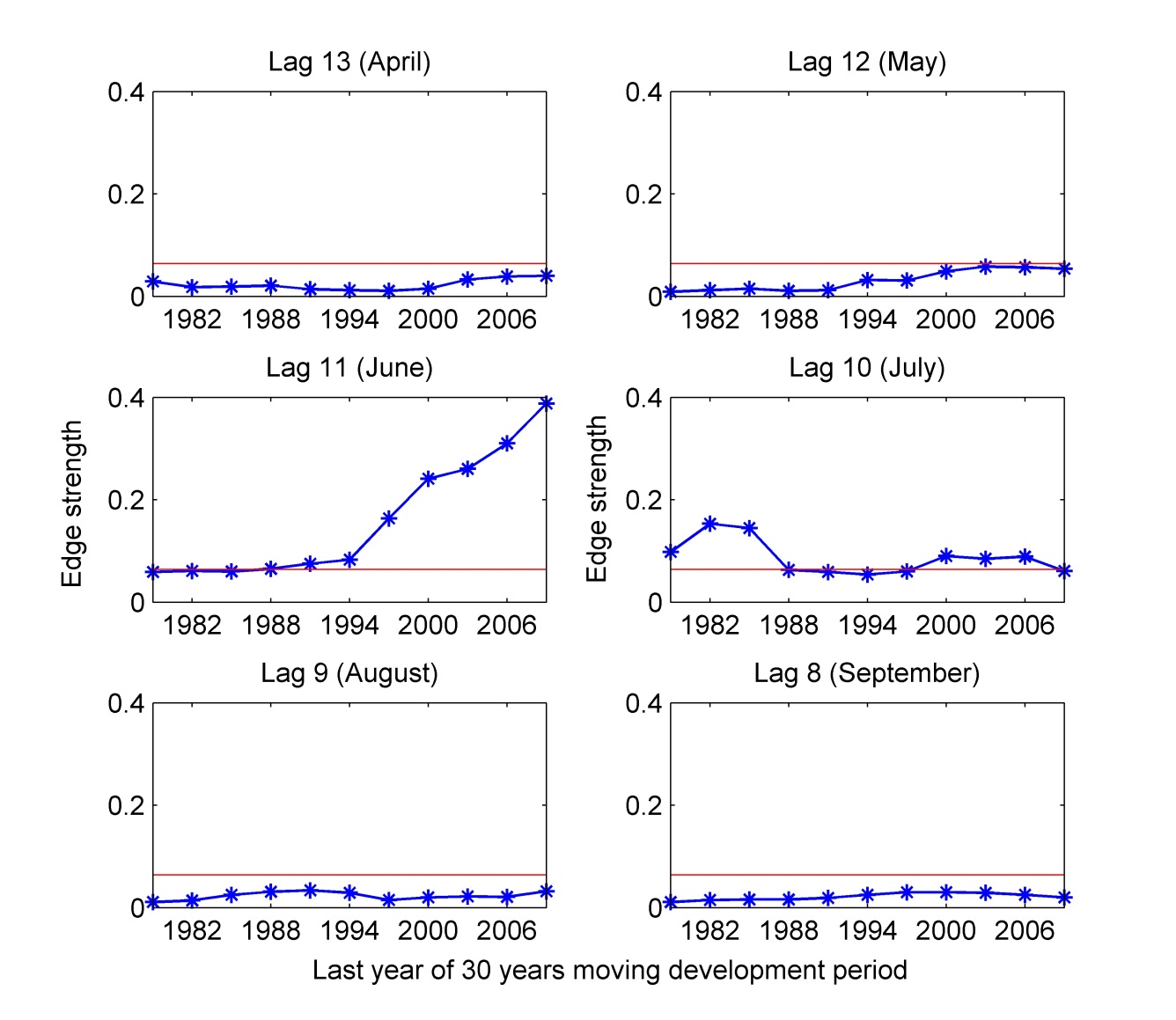


Figure S3: Time variation of edge strength between ISMR (predictand) and lags 8 to 13 of EQUINOO (predictors) obtained from the conditional independence structures along with significance threshold value (red line).


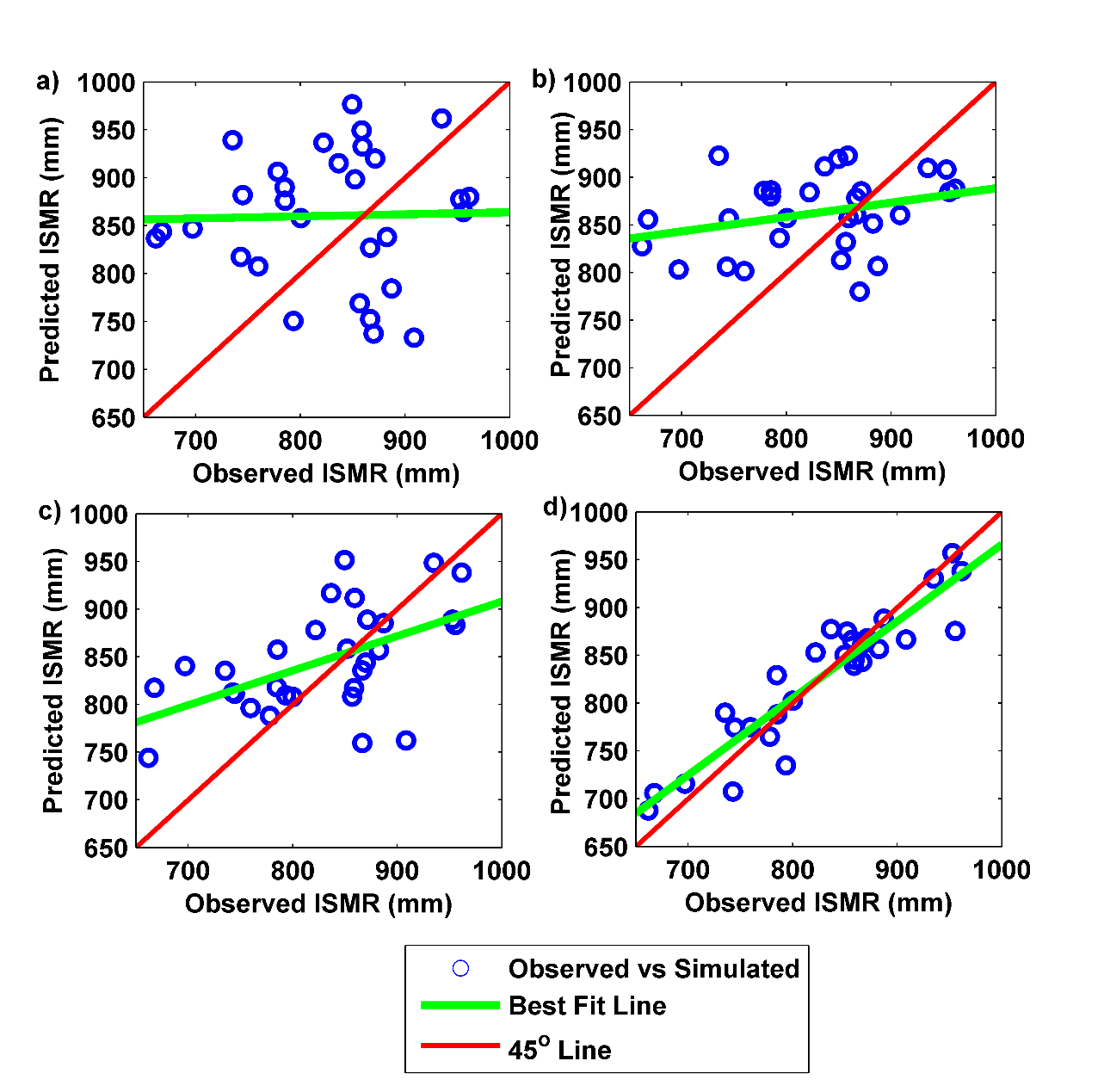


Figure S4: A scatter plot between the actual and predicted ISMR. (a) The plot obtained using the time-invariant SVR approach shows the most inferior performance, (b) The plot obtained using the time-invariant C-Vine approach also shows very inferior performance as the model is static in nature, (c) the plot obtained using time-varying SVR approach shows a comparatively better performance as the time-varying association of the climatic indices and ISMR is considered, and (d) time-varying C-Vine approach show the most superior results as vine copula rightly captures the complex association between ISMR and the climatic indices.
